# London's Problem Solved

**Published:** 1887-12-03

**Authors:** 


The Hospital.

### LONDON'S UNSOLVED PROBLEM:

### Its Unemployed and Casual Poor.

### NOT PREACHING BUT PRACTICE.

DURING recent successive winters the inhabitants of London have been amused, puzzled, horrified, and alarmed in turn. They have had forced upon their attention, through the Press and by gatherings of the unemployed and others in public places, the fact that there was in their midst—not in one district, but in all districts—a vast number of families and individuals without work, without homes, and without the means of subsisting from day to day. In previous winters attempts have been made to do something in a more or less systematic way by religious organisations, stirring addresses from the pulpit, devoted labours amongst the poor, and by public subscriptions to a Mansion House Fund which realised £75,000. These movements, so far as their results are ascertainable, have not mitigated the evil, or even temporarily alleviated it, but rather, on the contrary, so far as can be judged by conjecture and impressions, widened and extended the distress in a variety of ways.

During the autumn of the present year, and since, numbers of the unemployed and homeless have congregated in Trafalgar Square, where they have been attracted in ever-increasing numbers owing to the action of certain humane and kindly people who have distributed food, alms, and tickets for free lodgings to those collected together. However well-intentioned and natural the action of those kindly persons may have been, it has undoubtedly produced a number of new evils by inducing idle and insubordinate children to leave their homes and seek a life of adventure in the streets. It has, moreover, attracted all that was most worthless and disreputable to the very centre of the town in search of largess or plunder, or both. This condition of affairs has, however, produced one excellent result. It has aroused and fixed the attention of Londoners and of the country on the central fact that at all times there exists in London an immense amount of chronic distress, due in no small measure to the fact that the metropolis, unlike other parts, has failed to establish any system to provide the requisite machinery for dealing with it in an adequate way. Many schemes and proposals are in the air, or have been formulated with more or less completeness; but, so far, no one has attempted to deal with the problem here presented as a whole, or to suggest a remedy which promises, if applied with persistence and devotion by the inhabitants of every district of the town, to secure the removal of the existing evils once and for all. In these circumstances, one of the three planks in the programme of THE HOSPITAL being philanthropy, we purpose to-day to attempt to supply the omission, and to formulate a scheme which is at one and the same time so wide, deep, and all-embracing as to prove adequate to the needs of the vast population concerned, and yet so simple in its details as to render it possible that every Parliamentary district, and ultimately the whole metropolis, may work it successfully at small cost and without delay or risk of any kind.

We have said enough to show that preaching and publicity have been tried without adequate result; and most thoughtful people will probably agree that much as these two agents have done indirectly, they have but led us to the point of practice which is in truth the first object of to-day. First, then, let us ascertain in what respects the circumstances surrounding the poor in London differ from the circumstances of the same classes resident in the provinces. Those who have had experience in both places will admit that in the provinces there is much greater cohesion between different classes of society than at present exists in London. In the former the affluent classes are often the principal employers of labour, and, in some degree, and certainly in a far greater degree than is the case in the metropolis, the condition of their employés is known to them. This knowledge, however superficial and incomplete it may be, enables those interested in the artizan and labouring classes to distinguish at a time of distress the genuine working man from the loafer and the idle. The large employers of labour take an active interest in the administration of local affairs, the local authorities in consequence are generally held in high regard by, and are in touch with, the people. To take some examples out of many within the cognisance of this journal, we would point to the fact that in Birmingham, in Newcastle and Sunderland, in the Potteries, and in Glasgow, the working classes contribute more to the hospitals than the whole of the other classes contribute on Hospital Sunday; and in some of these instances more than all the other classes contribute in subscriptions besides. In London we know that the efforts made to secure the support of the working-classes to the hospitals have produced but a tithe of such contributions as we have referred to, and it cannot be said that the local authorities are held in the same high estimation by the people. The jurisdiction of the Corporation of the City of London extends over only a small area, and its members are selected almost wholly by non-resident ratepayers. Hence that authority, owing to its peculiar position, can directly know but little of and do but little for the people. The works outside the Corporation are in the hands of the district Boards of Works; the members of these boards are not elected by the people, and hence their actions are little heeded by the inhabitants of the districts over which they exercise control. The Metropolitan vestries come next, but as their powers in the matter of works have been usurped by the district boards, their duties are mostly confined to the management of local affairs. It is a matter of paramount importance that work of some kind should be found for the unemployed, to enable men who are willing to labour to be kept from the demoralising influence of charity, whether in the form of alms or of poor relief; and at the same time to remove all just cause for agitation and disturbance. In these circumstances, when distress is prevalent, the well-affected feel themselves compelled in self-defence, for want of personal aid and friendly influence, to join their forces to those of the disaffected, because agitation seems to offer them the only way by which public attention may be aroused, and practical help and sympathy secured. This is undoubtedly the position of affairs in London to-day, and it is one which calls for the attention and demands the personal devotion of every inhabitant.

### EXISTING AGENCIES FOR RELIEVING THE POOR.

### A Humiliating Confession.

Some of our readers will probably say after reading the introductory remarks on the previous page, you are overlooking the fact that we have in London a complete system of poor-law relief, and an army of charities supported by voluntary contributions which are the pride of the Empire and the wonder of the civilised world. Of all such we would inquire, Have you examined into the administration of the poor-law system in London, and are you in a position to state how far the numerous charitable agencies fulfil their functions, and do not in a measure defeat their individual aims by overlapping each other's work? We confess frankly that it is because we have found ourselves face to face with these questions without being able to answer them satisfactorily that we are induced to bring this whole question to the notice of the Press and the people of London, in the hope that something adequate may be attempted without delay. In the following pages will be found much reliable information concerning the poor law and its administration in the metropolis. The position of the unfortunate who is resident in London without work, without friends, and without funds, is truthfully represented, and such general information as is available is given of the position occupied by the charities of all kinds in regard to this question. We have dealt with the problem under two main heads to enable us to show clearly (1) the existing agencies, what they accomplish, and where they fail; and (2) what remains to be done. Every statement is made with the full sense of responsibiltiy, and has been checked by reference to official documents and sources of information. The proposed remedy is the result of many years' study of the questions involved, including a personal inspection of the methods adopted by American and foreign communities and the experience gained in this country by the action taken in times of special emergency like the Lancashire cotton relief fund, the history of which supplies instructive reading for Londoners to-day.

### I.—The Voluntary Agencies.

We have said in another place that the voluntary charities are the pride and admiration of the British Empire and the wonder of the world. The position of London as the capital city of the empire necessarily causes the friendless, the helpless, the infirm, the incurable, and sometimes even the acutely ill—the aged, the orphans, the unprotected, and the prodigal, when local help fails, to turn with a not unnatural instinct to one of the many charities which has its headquarters in London. It is necessary to recall this fact before we proceed to give the statements of the various voluntary agencies in London, the work of which tends to mitigate distress and relieve sickness, and to make provision for the aged, the helpless, and the orphan. That is to say, everyone must remember that the whole of the income of these various agencies is not disbursed upon purely local, that is, metropolitan cases. But although it is impossible to ascertain the amount of relief given to other than metropolitan cases, there can be no question that the larger portion of the income goes to relieve residents within the metropolitan area, or a limited radius beyond it. Omitting from the account missionary and book societies, and church and chapel building agencies, of which there are so many, the following is a complete list of the metropolitan relief agencies, compiled from the "Classified Directory," with the income they had at their disposal for the year ending 1885-6:—

### The Available Income in 1885-6.

47 *Institutions for those Permanently Afflicted, viz.* :—27 for the blind, 8 for the deaf, 9 for incurables, 3 for idiots—£198,436.

187 *for Sick Relief, viz.* :—93 hospitals, 48 general and provident dispensaries, 2 institutions for vaccination, 6 institutions for surgical appliances, 45 convalescent homes, 15 nursing institutions—£655,380.

158 *Pensions and Institutions for the Aged*—£442,685.

109 *General Relief, Food, and Loan Charities, viz.* :—92 institutions for general relief, 17 food institutions and loan societies—£390,702; 94 *voluntary homes*—£159,080; 6 *Orphanages*—£174,942; 74 *institutions for reformation and prevention*—£70,993; 45 *institutions for social improvement*, £69,884. Grand total of institutions, 758—Income, £2,162,102.

The work of most of the above charities can be readily conceived from the title given to the group under which they are classified, but a few words of explanation may be added in one or two instances. Thus the voluntary homes include institutions for ladies, women (principally girls), men, and boys (principally shoeblacks), infants, and night refuges for both sexes. The institutions for reformation and prevention include certified reformatories and industrial schools for boys and girls, and agencies for the fallen as well as for discharged prisoners of both sexes. Those for social improvement embrace employment agencies for ladies, women, men, boys, and others; and societies for apprenticing, emigration, general improvement, improved dwellings, and temperance. The sum represented by the aggregate incomes is very considerable, no doubt, and the combined good effected by its expenditure under the existing system must be very considerable. At the same time, we have at present no means of knowing to what extent the work of one institution overlaps that of another, or how far the unworthy take advantage of the voluntary relief offered by seeking the aid of one, or, in the case of hardened offenders, of a dozen or more, in the course of the same year.

The facilities at present afforded to worthless and designing people who will live upon charity if they can, are the weak spots in the armour of metropolitan charity to-day. Attempts have often been made to prevent this overlapping in the case of whole groups of charities such as hospitals, and at the present time a committee is striving to organise a system with a view of permanently preventing it. The managers of most charities feel more and more that the claims upon it are increasing year by year to an extent out of all proportion to the means placed at their disposal. We doubt not they would gladly welcome, and willingly agree to co-operate in bringing to a satisfactory conclusion, any scheme which offered them reasonable facilities for protecting themselves against abuse by the unworthy. From the wider point of view of the greatest good for the greatest number of the inhabitants of London, there can be no question that to secure an adequate remedy for the chronic distress which must ever exist, any sound proposal which offers practical assistance by preventing waste in the overlapping of the distribution of charitable funds should be adopted. We believe that an appeal to every relieving agency, every Church, and every charitable person to help not only to work for the poor, but to work in co-operation, to become parts of a great voluntary system which would provide adequate relief for all in need, whilst ignoring none, though it might be unanswered by some for a time, would ultimately commend itself to the adoption of all. Such a system of co-operation as is here suggested expresses the most beautiful idea of modern times, and one which has found practical expression in the Hospital Sunday Fund organisation, where all co-operate together as associated charities and associated churches, whilst retaining their separate rights, and life, and work. We trust the day is not far distant when the metropolitan charities will embrace this idea as churches of all denominations throughout the English-speaking world have already done, for then their work would be doubly blest, because it would not only relieve the necessitous, but would prevent wasteful and useless expenditure on the unworthy and the fraudulent. We have prepared a scheme to enable every constituency to do this work readily and effectually, and we propose to publish it in an early issue.

### II.—THE METROPOLITAN POOR LAW SYSTEM.

The poor law system as it exists in London at the present time will now be considered. We propose as briefly as possible to explain the system as a whole, its administration and executive government, the method upon which out-door relief is at present given, and also the dietary treatment of the inmates of the metropolitan workhouses. Having done this, we shall conclude by pointing out how far that system provides for the unfortunates who may find themselves in London without work, friends, or funds. First, let us pause for a moment and inquire what is

### THE PAUPER POPULATION

of London to-day. The estimated population of the metropolis in 1874 was 3,356,073, and in 1886 it had risen to 4,083,928. In the first year the mean number of paupers of all classes was 115,010, and in the last year 101,443. In other words, the ratio of paupers per thousand of the population was 34·1 in 1874, and 28·4 in 1886. It is satisfactory to know that the ratio of paupers to the population has steadily decreased year by year, but this will be seen on reference to the table in another column. Here we may point out, however, that although the population had increased by upwards of half-a-million in the year 1886 as compared with the year 1874, the paupers had fallen by nearly 14,000 souls, although the number of indoor paupers had increased from 39,165 in 1874 to 54,583 in 1886. This increase in the amount of the indoor relief afforded was no doubt due to the alteration in the system of outdoor relief, the number of outdoor cases having been 75,845 in 1874 and 46,860 in 1886. Thus the ratio of indoor paupers per 1,000 rose from 11·6 in 1874 to 13·4 in 1886, whilst that of the outdoor paupers fell from 22·5 per thousand of the population in 1874 to 11·5 in 1886. or to put it another way, the cost per head per pauper in 1874 was £14 4*s*., and in 1886 it was £22 5*s*. 2¼*d*., an increase of upwards of 33 per cent. We must also direct attention to the cost per head on the mean number of paupers of all classes in the West of England and Wales, as compared with that in the metropolis. The cost in 1874 in Wales and the provinces was £9 5*s*. 2¾*d*. per head, and in the metropolis £14 4*s*. Whilst in 1886, the cost in the former case was £10 12*s*. 6¼*d*. per head, whereas it amounted to £22 5*s*. 2¼*d*., or more than double in the metropolis. This is a matter which urgently demands attention, because the improper or extravagant administration of poor law funds in the metropolis, if it can be prevented, must free some hundreds of thousands of pounds, which could be usefully expended in aiding the unemployed and distressed poor to maintain themselves and their families in respectability and comfort. It would be foreign to our present purpose to press yet more forcibly, as we might do, the lessons of this table; and we must content ourselves with pointing out to the ratepayers this one crowning fact, that although the pauper population has so greatly decreased, and although the rateable value has risen to the extent of 50 per cent., the rate in the pound for relief which they have to pay has only fallen from 1*s*. 7¼*d*. in 1874, to 1*s*. 6½*d*. in 1886. If the present discussion does nothing more than attract and fix public attention upon the administration of poor law expenditure in London to-day, it will have accomplished much in the interest of all classes of the community.

### WORKHOUSE MIS-GOVERNMENT.

There is an equal need of reform in the executive management of the Metropolitan Workhouses. This is not surprising

### THE METROPOLITAN POOR-LAW EXPENDITURE AND PAUPER POPULATION, 1874-1886.

THE FOLLOWING TABLE SHOWS THE RATEABLE VALUE, THE AMOUNT OF RELIEF TO THE POOR, THE ESTIMATED AND PAUPER POPULATION, THE COST OF IN-DOOR AND OUT-DOOR RELIEF, AND THE COST OF PAUPERS OF ALL CLASSES IN ENGLAND AND WALES AND THE METROPOLIS, FOR THE TEN YEARS ENDED 1886, COMPARED:—

| YEAR. | RATEABLE VALUE. | TOTAL RELIEF TO POOR. | RATE IN THE £ FOR RELIEF.* | ESTIMATED POPULATION. | MEAN NUMBERS OF PAUPERS OF ALL CLASSES. In-door. | | Out-door. | | TOTAL IN-DOOR AND OUT-DOOR PAUPERS. | | COST PER HEAD ON MEAN NUMBER OF PAUPERS OF ALL CLASSES. England and Wales. | Metropolis. |
|---|---|---|---|---|---|---|---|---|---|---|---|---|
| | | | | | Mean Number. | Ratio per 1,000. | Mean Number. | Ratio per 1,000. | Mean Number. | Ratio per 1,000. | | |
| | £ | £ | s. d. | | | | | | | | £ s. d. | £ s. d. |
| 1874 | 20,746,458 | 1,633,182 | 1 7¼ | 3,356,073 | 39,165 | 11·6 | 75,845 | 22·5 | 115,010 | 34·1 | 9 5 2¾ | 14 4 0 |
| 1875 | 21,124,179 | 1,588,709 | 1 6½ | 3,400,701 | 39,882 | 11·6 | 69,438 | 20·3 | 109,320 | 31·9 | 9 7 0 | 14 10 7¾ |
| 1876 | | 1,618,822 | 1 6½ | 3,445,160 | 39,704 | 11·4 | 58,858 | 16·9 | 98,562 | 28·3 | 9 15 9½ | 16 8 5¾ |
| 1877 | 23,444,876 | 1,695,599 | 1 5¾ | 3,489,428 | 41,862 | 11·8 | 52,574 | 14·9 | 94,436 | 26·7 | 10 5 7 | 17 19 1¼ |
| 1881 | 27,544,446 | 1,907,155 | 1 6¼ | 3,769,390 | 50,175 | 13·3 | 48,863 | 13·0 | 99,038 | 26·3 | 10 4 10 | 19 5 1¾ |
| 1882 | 27,973,978 | 2,090,753 | 1 6¼ | 3,829,751 | 51,136 | 13·3 | 49,188 | 12·8 | 100,324 | 26·2 | 10 8 10 | 20 16 9½ |
| 1883 | 28,438,385 | 2,172,294 | 1 6¾ | 3,893,272 | 52,157 | 13·4 | 50,038 | 12·9 | 102,195 | 26·3 | 10 13 6 | 21 5 1½ |
| 1884 | 28,954,020 | 2,267,330 | 1 7¼ | 3,955,814 | 52,979 | 13·4 | 46,446 | 11·7 | 99,425 | 25·1 | 10 19 5 | 22 16 1 |
| 1885 | 29,410,489 | 2,418,049 | 1 8 | 4,019,361 | 54,610 | 13·6 | 45,603 | 11·3 | 100,213 | 24·9 | 11 0 10½ | 24 2 7 |
| 1886 | | 2,258,029 | 1 6½ | 4,083,928 | 54,583 | 13·4 | 46,860 | 11·5 | 101,443 | 24·8 | 10 12 6¼ | 22 5 2¼ |

* The rate in the £ is calculated on the rateable value according to the Valuation Lists in force at the beginning of each parochial year.

### POOR LAW EXPENDITURE AND MANAGEMENT.

The above table is worthy of the close attention of every intelligent ratepayer throughout the metropolis. It reveals a state of affairs which calls imperatively for inquiry, and anyone with any knowledge of administration, or any regard for economy, cannot but be struck with the facts which it brings out for their information and guidance. We have shown in the foregoing statement how much the pauper population has fallen off during the last ten years in point of numbers, yet the total expenditure upon poor relief has increased from £1,633,182 to £2,258,029; in other words, it cost nearly £600,000 more to relieve 101,443 paupers in 1886 than it cost to relieve 115,010 in 1874: considering the evident mismanagement with regard to their administration which we have already shown to exist. Those who read to the end will become aware of the evils and insufficiency of the casual ward accommodation, and we shall therefore confine our attention now to the workhouses and poor-law infirmaries. As structures, the poor-law infirmaries, which have for the most part been built since the passing of Hardy's Act in 1866, leave little to be desired, and they are more complete and better adapted to this purpose than more than one of the voluntary hospitals; yet the poor are shy of entering them, and have a wholesome dread of the ill-repute consequent upon a residence within their walls. The workhouse system has polluted the atmosphere of the poor-law infirmaries, and the social leprosy, which a residence in either entails, is happily repugnant to the feelings, as well as opposed to the moral sense of the respectable portion of the poorer classes in London. This fact gives us the key to the defects of the management of the metropolitan workhouses.

Mismanagement or Worse!

Necessarily all classes, and consequently the lowest and most abandoned classes of the community, have to be provided for in poor-law establishments. It is an appalling thought, and should be a surprising fact, that the intelligence of this generation has not yet displayed a sustained and successful demand for the classification of the inmates of workhouses and poor-law infirmaries. The consequence is that the present system of administration of indoor relief to the pauper population is mainly a system of repression that is a penal system, and not an educational, and therefore a provident and beneficial system, as it ought to be. Metropolitan guardians, as the report of the Local Government Board eloquently testifies, are prepared to supply the poor with improper food, and to improperly expend the ratepayers' money without regard to value received or ordinary business principles. If this is regarded as a strong statement we would refer ratepayers to the fourteenth report of the Local Government Board, pages 165—168, where the average price paid for various articles used in the Metropolitan workhouses is given for the first time. They will find that flour varies in price from 40*s.* to 26*s.* 6*d.* per sack; bread from 14*s.* to 9*s.* per cwt.; butter from 154*s.* to 72*s.* per cwt.; cocoa from 130*s.* 8*d.* to 42*s.* per cwt.; arrowroot from 112*s.* to 28*s.* 8*d.* per cwt.; potatoes from 126*s.* 8*d.* to 66*s.* per ton; wine from 14*s.* 11*d.* to 5*s.* 3*d.* per gallon; and ale from 52*s.* to 25*s.* per barrel. Such differences in price mean one of two things—gross mismanagement or fraudulent patronage; and it is for the ratepayers to decide by investigation which is the true explanation of the extraordinary difference in the prices paid for the same article. The guardians, however, that considerations of economy prevent classification in individual workhouses; and so the really deserving poor would sooner starve to death in an obscure corner than enter a Metropolitan workhouse, and be there compelled to mix with the scum of the population. If London had one municipality, then classification would be easy, because particular workhouses could be devoted to the reception of one class only of the pauper population; and an educational, as opposed to the present repressive system, would be enforced, with the happiest possible results. Of course there is no reason why classification should not take place in the existing workhouses, but to secure this end Londoners must arouse themselves, each in his own locality, and bring pressure to bear upon the guardians persistently and consistently until this necessary reform becomes an accomplished fact.

### THE FEEDING OF THE POOR IN WORKHOUSES.

In any considerable aggregate of human beings there will always be some who, for various reasons, will not gain a subsistence by their own exertions. They will fall below the general level, and if left to themselves will either starve, beg, or thieve. These people must always be dealt with by the more successful part of the community; and it is open to the latter to deal with them in one of several ways. In the first place, they may be simply left alone to live as they can —partly by working, partly by begging, partly by stealing. Or it is quite at the discretion of a sufficiently tough-hearted community to pass an edict for the summary execution of all aged and incapable persons in order to avoid the burden of their maintenance. Or, in the third place, the more prosperous may resolve to take upon themselves the responsibility of the care and support of the incapable. In this country we have elected to recognise that the poor have certain claims upon us, and we have admitted those claims to the full extent of taking upon ourselves the whole burden of their care and maintenance, when there is sufficient proof that they are no longer able to support themselves. That being so, two or three questions of very great importance should be asked and answered by each person who wishes to do his duty fully and intelligently in the matter. These questions have reference to the feeding, clothing, housing, and general management of the class under consideration. At present the subject of feeding may well engage our whole attention. How are paupers fed? How have they been fed? How should they be fed? To begin at the beginning, we may state some facts from the history and experience of past but still recent times. In England the pauper population has for many years been fairly well housed and not ill-clad. The poor-houses, which fifty years ago were, in many cases, filthy and loathsome pesthouses, both physically and morally, have almost universally given place to well-built, well-arranged, and well-conducted institutions, highly creditable to the parishes and the country at large. The feeding of the poor cannot be spoken of in terms of such unqualified approbation. No one expects, not even the paupers themselves, that the table of the union-house shall resemble that of a City company in its civic banquetting hall. But what we who pay the rates do expect, and must insist upon, is that they shall have food of such quality and quantity as shall maintain life at its full natural vigour. Falling short of that we simply decree the gradual death of the pauper by slow starvation, rather than his immediate extermination by poison, hanging, shooting, drowning, or other prompt, speedy, and inexpensive method.

How they Live in the Workhouse.

Food then of such quantity, quality, and variety as is required for the maintenance of the normal strength of the individual is the irreducible minimum. An examination of the diet tables of a large number of workhouses will show how far they have departed from any such standard as this. Taking the daily meals in order, for *breakfast*—at sixty-five workhouses, selected indiscriminately from several counties —the quantity of bread supplied to each able-bodied man varies from 5 to 8 oz., and to each able-bodied woman from 4 to 6 oz., and to this is added from 1 to 2 pints of milk (skim milk) for men, and a rather smaller quantity for women. In some cases porridge or gruel may be substituted for the milk; but it is important to know that the porridge consists of oatmeal and water in the proportion of 1 or 2 oz. to the pint, with sometimes a little flour and milk added. Gruel is also prepared in a similar way, and is practically of the same nutritive value as the porridge. Such the average breakfast of the able-bodied pauper all the year round. The changes are rung on bread and milk, bread and gruel, bread and porridge, or they may be placed in any other combinations the reader pleases, only remembering that such combinations do not alter the facts, or the variety of the breakfasts. *Dinner* is of course a rather more substantial meal, and includes either solid meat or soup, with bread and some vegetables. The quantity of cooked meat free from bone supplied at dinner to male adults varies from 4 to 6 oz. In most houses women have 1 oz. each less than men. The quantity of potatoes or other fresh vegetables varies from 7 to 20 oz—16 oz. may be taken as the average. To the 7 oz. of potatoes a small portion of bread is added in many cases, but no bread where the average is higher than this. Taking 5 oz. as the average of meat and 16 oz. as the average of potatoes, vegetables, and bread, we get 21 oz. of more or less solid food as the supply for dinner. Thi represents the best kind of dinner provided, and usually is not supplied oftener than twice a week. On one or two days soup or hash with bread, and on others bread and cheese, or suet dumpling, or rice pudding may take its place. It sometimes happens that "rice milk" is the staple dish for dinner, though the report is careful to state that this mixture need not necessarily contain any "milk." In selecting the solid meat dinner we have given the *best* of the paupers' mid-day meals. The "rice milk" day must surely be the worst, especially when the "milk" is wanting. *Supper* follows dinner, tea being omitted. The supper is practically a repetition of the breakfast, and consists of bread and milk, or bread and "porridge," or gruel, made of course as before described. The quantity of bread varies from 6 to 8 oz.

Putting these three meals together, we get a daily average of solid food for each adult able-bodied man of 34 oz. for the best days, and for the rice milk and other bad days a much diminished quantity. The women, as has been shown, usually have a smaller portion of each article of diet. The aged, the infirm, and the infants are treated separately, and are much more under the immediate control, as to diet, of the resident or visiting medical officer.

The general effect of this dietary is said to be such as to maintain in the inmates of the house a fair degree of health and strength, but by no means to please the palate or the judgment. The latter statement we can quite agree with, but not the former. In our experience, the adult pauper is a feeble-bodied, nerveless, and melancholy creature, and this condition we regard as being due principally to the small quantity, poor quality, and inadequate variety of his food. The report admits that for the aged and infirm, but especially for infants and growing boys and girls, the workhouse dietary is eminently unsuitable in many cases, and leads to feebleness of constitution and defective physical and mental development. It is to be borne in mind that most of the articles of daily consumption in union-houses are purchased by tender, and that, as it is generally known that the lowest tender will be taken, the poorest kinds of meat, bread, vegetables, milk, etc., are generally supplied. Now, if it be noted that the average adult working man in his own home consumes nearly 40 oz., or about ¼ lb. more of solid food daily than the pauper, and a correspondingly increased quantity of milk, which is new and not skimmed; and if it further be remembered that the poor man or his wife purchasing for the family has a discretionary choice as to the quality of food she will purchase, it is seen that the nutritive efficiency of the pauper's diet is little, if any, more than two-thirds of that of his fellow-workman who is maintaining himself. In certain cases known to the writer, the labourer, when fed at his employer's expense, as in Yorkshire farm houses, will habitually eat from 50 oz. to 60 oz. or more of solid food daily, fully a third of which will consist of flesh—meat without bone. This class of men, as was shown by the late Professor Sir Robert Christison, not only eat more, but are capable of doing more work than any other class in Great Britain. It is undoubted that, in many instances at least, paupers are underfed, and it is equally undoubted that underfeeding is slow starvation, and produces premature decay and death. As the object of receiving paupers into union-houses is neither penal nor correctionary, but simply for their maintenance, it is both rational and humane that they should have such a quantity, quality, and variety of food as will sustain their strength at its normal level of vigour.

### CASUAL WARDS AND THEIR OCCUPANTS.

One out of every forty persons in London is a pauper—that is, out of the four millions that inhabit London there are 100,000 who are in receipt of parochial assistance. This estimate takes no account of the relief afforded by private charity, either in an organised form or otherwise; it includes only those who are a direct burden on the rates, either as inmates of workhouses or recipients of outdoor or casual relief. The number of course varies from season to season; in winter it is larger than in summer, and slackness of trade from any cause tells indirectly on the whole mass of the population, and drives many, hitherto independent, to seek for a time at least, parochial relief. We have heard a good deal of these lately. "The Unemployed" have become a weariness to the flesh, so often have we been told of their wrongs and their sufferings, so often are we assured that "something must be done for them." Assuredly, something must be done; some provision must be made for these houseless and starving fellow-mortals; but what is that provision to be? There's the rub! Our charitable instincts are checked by the assurance that promiscuous giving only encourages improvidence and laziness; on the other hand, we all feel that a little money is well invested if it enables some unfortunate, but honest and hard-working fellow-creature, to tide over a few weeks or months till the opportunity of earning a living shall return, and we can thereby save him from becoming a permanent burden on the rates. This is the primary idea that led to the establishment of casual wards—to provide food and shelter for the temporarily starving and homeless, till they should have an opportunity of becoming again self-supporting. From this point of view the casual ward is an institution more important than even the workhouse; the latter is, speaking generally, the last refuge of the infirm and useless, the wrecks stranded on the shores of life; the former should be merely "a port in a storm," and should be connected with some means of enabling those who avail themselves of its shelter to seek their way back to independence, and, if possible, prosperity. The demand for such shelter will of course be exceedingly variable, and it is doubtful if it would be possible to provide sufficient accommodation for a strain like the present, but we cannot but see that what is given is utterly inadequate. In all the casual wards of the metropolis the number of berths is 1,519, of which 956 are reserved for men, 474 for women, and 89 for children. It may safely be assumed that there are more than 1.500 people who seek a lodging for the night in London, and as the wards are filled up pretty much on the simple principle of "first come, first served," those who apply late in the evening are often enough refused admission. But a counterbalancing advantage is the early hour at which applicants are allowed to enter, the door being opened at four o'clock in the afternoon between October and March, and at six during the summer months. Thus those who are weary and hopeless of work for the day may find food and shelter as soon as night falls. And who are the people who take advantage of the accommodation provided by the State in the form of casual wards? The narrative of a visit we paid to one a few days ago will be the best answer to that question.

### THE CASUAL WARD IN BAKER'S ROW.

It was about eight o'clock in the evening when we turned out of Baker's Row to enter the casual ward of the Whitechapel Union. Entering a small, brightly-lighted corridor, we introduced ourselves to the superintendent, whose office was formed by a glazed partition in one corner that stretched half way to the roof. Entering this compartment, we found the superintendent at work on a task which assuredly the immortal Bumble would have counted beneath the dignity of his office—namely, cutting a loaf into slices by means of a sort of beneficent guillotine that stood on the table. These slices would hardly have been suitable for serving in a drawing-room at afternoon tea, for they were about an inch and a half in thickness, and were brown in colour; but the bread seemed clean and wholesome, and the size of the portions would be no disadvantage in the eyes of hungry men and women. As it was still early in the evening, the wards were not yet full, but a sufficient number had been admitted to promise but little accommodation to any wh should apply at midnight or in the small hours. Already 34 men and 18 women had been admitted, and the total accommodation provided numbered 45 beds for men and 25 for women. The names of those who profit by the institution are duly recorded, along with their ages and birthplaces, and in the case of those who had come in the previous night, and had therefore been made to spend the day in the workhouse, there was also noted the task that had been set them, and the proportion of it that had been done. Of these only a few had been employed in the regulation oakum-picking; most of the men had been carrying coals in the workhouse, while the women had been engaged in cleaning. Of the oakum-pickers, one only, so far as we could see, had done his allotted 4 lb. One had developed such an admirable quality of idleness that he had got through only 1¼ lb.

### Where the Inmates came from.

Looking over the register of that night's admissions, we observed that the greater number of the applicants were natives of the East-end; Ireland supplied a fair quota, which was pretty nearly balanced by those from the provinces, and there was one Scotchman. "That's one too many!" exclaimed one of us who hails from over the Tweed. "We have very few Scotch," said the superintendent reassuringly. "Does this one drink?" was the next question, the national vice of North Britain being the first cause of destitution that suggested itself to the Commissioner's mind. "No," was the answer, "he is deformed, and has got out of work. He's not one of the 'regulars.' I know most of those that have come in to-night; but he's a new-comer." A subsequent conversation with the Scotchman gave fuller details. He was a cabinetmaker; and the firm he worked for had been obliged to dismiss a number of men, as, owing to the competition caused by the importation of German workmen their trade had declined. He, as one of the weakest in the workshop, had been among the first to be dismissed. It says little for our modern taste and culture that the general complaint at present is that there is little demand for well-made furniture, and that therefore good workman, who cannot turn out well-finished goods at the rate at which inferior work is done, are those who suffer most. All departments of upholstery are at a low ebb just now; and among women, those who used to make good wages as carpet-sewers are among the most destitute.

### Their Antipathy to Foreigners.

But these Germans and foreigners generally, how they are hated by the unemployed Englishman! While we were talking to the superintendent there entered an applicant for the shelter of the ward. He was, it seemed, a baker, and had been out of work an indefinite number of months —he couldn't exactly tell how long. He was altogether a vague and indefinite individual, who thought he could do "anything in the labouring way," but had not yet begun to seek for employment outside his own trade, nor even very vigorously within it. But he was quite decided on two points—first, that Free Trade was ruining us, and secondly, that "them foreigners" ought to be put down. "But why?" he was asked. "Is there anything against them but that they can live on less than Englishmen, and do as much work? That's no disgrace." The ex-baker looked disgusted, and proceeded to speak in disparaging terms of the foreigner's food. "They'll make themselves some mess with haricot beans and a little bit o' bacon, and be quite contented with it," he said; and, disregarding a protest from the Commissioners that they found haricot beans not only wholesome, but palatable, he went on to another grievance against the Germans—"that they didn't mind working from one week's end to another without any break, except taking out their young lady on Sundays." All these things he heartily despised.

Without sharing the ex-baker's contempt for foreigners, we must admit that their competition in the labour market is in many respects dangerous to our fellow-countrymen. The employer of labour naturally chooses the cheapest workmen he can get, and these in almost all cases are foreigners. The German clerk who can "talk six languages, work fifteen hours a-day, and live luxuriously on a pound a-week," is no myth, but a living personage, against whom the British-born cannot compete; and in all departments of industry the same dangerous rivalry is present. Among the present causes of distress not the least is the constant immigration of Germans and Jews of many nationalities. When they succeed, they take from Englishmen the work the latter would otherwise obtain; when they fail, they aggravate the existing want. Whether our ex-baker fully grasped the meaning of his statements is doubtful. He enunciated general principles certainly, but all with a special application to his own case; implying thereby that no blame attached to him for being out of work, and that no blame could attach to him if he never had work to do again. Meanwhile he seemed to be not much troubled about his position; certainly to feel in no way humiliated by it. He was contented; in spite of these uncomputed months of idleness he seemed well-fed; "he got along somehow," was the only explanation of his mode of living that he would vouchsafe. It was evidently mysterious, with that gloomy mystery to which the police can sometimes furnish a clue.

### What made them Casuals.

He was not so candid as most of the men in the ward, who admitted that they had come there through their fondness for "a drop o' drink." Possibly this is the class which most largely fills our casual wards. There are some who, owing to simple misadventure, have lost their situations; who are the victims of those fluctuations of trade which it is beyond the power of the workman to regulate, and who have neither skill nor opportunity to fit themselves to earn their living in another way than that they have been used to. There are many who have never been anything but casual labourers, remaining in that condition by reason of general incompetence. When they have work to do, they do it badly; those who work well go by natural selection into the class of the permanently employed, at regular, though low wages; but of the large remnant, what the dock contractors and others who employ them know best is that they take twice as long over their task as the permanent workmen, and therefore they have no chance of being employed except in the occasional seasons of special strain. But a great many have lost the opportunities they might have had through this tendency to drink. That they should drink is in no way strange. Without going into the familiar temptations that beset the working classes, it may be remembered that many, such as stevedores and other dock porters, earn considerable wages at the price of very severe efforts, and consequent exhaustion. This naturally arouses a desire for the stimulation of alcohol, and leaves them, even when they are making from 15s. to 20s. in a very short time, a poor class. But comparing the amount of employment afforded with the number of men who seek it, we perceive at once that there must always be a large number of men more or less constantly among the ranks of the unemployed. To give only one instance from the serried ranks of statistics which have been marshalled on this subject, we find that the employment afforded by the docks in the Tower Hamlets never demands much more than 6,000 men, and at slack times not more than 1,500, while it is counted that 10,000 men rely chiefly on this labour for their living. This implies that there must always be at least 4,000 unemployed men in connexion with this industry alone—not of course the same men continually; he who gets work to-day may miss it to-morrow, and *vice versa*. Many of these, being more permanently unfortunate, through lack of strength or other defect, will drift into the casual ward, and thence perhaps into permanent pauperism.

Some of the Inmates.

To return, however, to the ward we visited. The dormitories to which we were conducted were long rooms dimly lighted by a gas jet high on the wall, and protected by a strong glass globe from the possible missiles of some rebellious spirit. The beds consisted of hammocks of cocoa matting, slung over hot-water pipes, and furnished not over-luxuriously with a grey blanket and a rug by way of bed-clothes. At the time we visited them the rooms were tolerably warm, but the general complaint was, that in the early hours of the morning, just when the cold is greatest, the pipes got cold, and sleep was almost impossible. About the quality of the beds there seemed no grumbling—indeed, they were by some considered to compare favourably with those in the common lodging-houses.

"It's better than the bed I paid fourpence for last night," said one old woman; "and at least you're left in quiet. You ain't 'wakened at three or four in the morning by people coming in drunk, and shouting and swearing in a way to make you shudder."

This comparatively grateful utterance came from one who was quite new to a casual ward—a charwoman, who to the age of sixty-six had "kep' herself and kep' herself re spectable," as she said, with some pride; but on whose power of work and opportunities for obtaining it age had began to tell. "The younger ones get the work when there's any to be got," she said, with a sigh. "Folks that know me will keep me on, but I can't get into new places; and now that my daughter's husband is out of work—I used to live with her—they can't spare a bite for me. There's a house I go to still one day every week; that's always something; but the hard times has been telling on the people I worked for too. There was one lady, a dressmaker, I went to for years; she told me a month or two ago she couldn't afford to employ me any more, and it's the same with others."

"Yes, I know that," put in another woman. "There's my sister; she keeps a laundry in a small way, and I used to work for her, but now she can hardly keep going herself, far less give me any work. And she says that she might keep a girl answering the door all day just telling women that she can't give them a day's work."

"How is it that things are so bad with her?" was asked.

"Oh, what with the riots and the fever, there's a lot of people haven't come back to town, and that affects every-body."

Complaints of the Treatment.

"Well," said the old woman, who had meanwhile been thinking over her own case, "it's something to have a place like this to come to when you've got no home and no money to pay for a bed. It's better than sleeping in the streets, any way."

"Is it?" put in a third. "Maybe it is; but you've got to pay for your bed here too. You're imprisoned here till you've worked off the price of your night's lodging; and you're starved besides."

"Surely not!"

"Well, I call it starvation—dry bread and gruel. I'm not afraid to speak the truth, and I say we're crushed and kept down, and starved and imprisoned—kept three days if we won't pick their oakum and scrub their floors."

Here was a grievance which we considered worth inquiring into. On inquiry it appeared that the food supplied to these casual paupers, as enacted by a general order in 1882, and amended by another in 1887, consists of 6 oz. of bread and a pint of hot gruel or broth for supper, and the same for breakfast. As payment for this and their bed, men are expected to break 2 cwt. of stones, pick 1 lb. of oakum, or work for three hours at digging, wood-cutting, or some similar task. Women were expected to pick half-a-pound of oakum or spend three hours in scrubbing or clean-ing. Naturally many of the applicants for casual relief object to this labour test, which is, nevertheless, essential, as without it the casual ward would offer a greater temptation to the lazy and improvident than it now does. There are, and always have been, plenty of people who would almost as soon starve as work, and it is natural that the ratepayers, who, whatever their station, are working men, should object to their money being expended in the support and encouragement of such a class as this. A labour test of some sort is absolutely necessary. Some parishes, which are not provided with casual wards have tried a system of free lodging tickets, for which no such payment in kind is demanded. Unfortunately, the result of this was to bring to these unions casual paupers from other parts of London, and even to attract persons of a similar kind from the provinces. It is not likely, therefore, that the experiment will be repeated.

How the Casual Pays for Accommodation.

According to law the guardians are authorised to detain every casual pauper two nights and a whole day in the ward, exacting from him or her nine hours' work at some of the tasks spoken of above. In the "Casual Poor Act" of 1882 it is also enacted that anyone who is admitted more than once within a month into the same union can be detained three days after his admission, and may be required to spend the time of detention in the workhouse of the union. These were the regulations of which the woman whose words I have quoted complained. It is right to state, however, that though in both the male and female wards there was plenty of chatter all the time the Commissioners were present, and the "casuals" were, almost without exception, ready to narrate their personal opinions and experiences, this is the only complaint of that nature which was made. Moreover, as the pseudo laundry-maid, who seemed to be an experienced casual, explained, these rules are now relaxed at the discretion of the guardians.

"Both at the City and Marylebone," she said, "they let us go as soon as we liked, in order to give us a better chance of getting a bit of work. They kept one or two of the women to help in the washing-up and cleaning, but all the others could go whenever they wanted."

On inquiry we find that this change is due to a circular letter from the Local Government Board, dated 16th April, 1885, the instructions in which are reiterated in another, dispatched so lately as 7th November, 1887, advising the Guardians to permit casual paupers to leave the wards as early as they chose, "especially in the case of casual paupers, who there is no reason to believe would be unwilling to work if work could be obtained by them."

"If Work could be Obtained by Them?"

But this is the real difficulty. The men and women are dis-charged to seek for work without guidance or influence to help them, probably to wander about helplessly through the day, and seek the shelter of another casual ward at nightfall. If a labour bureau could be established in connexion with these wards, where those who lodged in them could be put in the way of obtaining work—real money-making work, which, however hard, would pave the way to independence and self-respect; not a mere "labour test," though that certainly is necessary under existing circumstances—if this were done, the casual ward would become more of a helpful institution and less of a tramp's hotel than it is at present. Then the industrious and unfortunate would receive the chance of a fresh start, while the idle would be shown in their true colours. In short, the organising and helpful work of real human charity would be joined to this mechanical "giving of relief." At present there is no classification of the applicants attempted. They come and go, and it is nobody's business to inquire into their character and capacity. By another system a process of natural selection could be instituted whereby the casuals themselves would be benefited, and probably in the end the ratepayers' pockets relieved. We do not pretend, however, that casual paupers are, as a class, people who will easily be elevated. Even in those who once were of a better type long periods of idleness and dependence have developed laziness and loss of self-respect; and some have never had any other desire than to get through life with as little labour as possible.

### The Matron's Opinion and Our Own.

"What is your opinion of your charges?" we asked the matron as she was showing us the warm and scrupulously clean bath-room, whose enamelled tubs and ample supplies of warm and cold water would have been welcomed in many a middle-class household; though it is probable that they are not appreciated among those who profit by them.

"Not a very high one," she answered. "There's good and bad among them—perhaps more that have been unfortunate at present than usual; but the greater number of them come to this through their own vices, and some of them are, you may say, born and bred casuals. My heart aches sometimes when I see mothers bringing their young children here, bringing them up to look upon these wards as their natural home."

Official utterances are sorely disparaged just now; but we venture to think that this opinion, though offered by a workhouse official, is both sincere and just. At least, recalling what we saw and heard in the casual ward of the Whitechapel Union, it represents our own impressions, with only this variation—that not only for the children, but for all who shelter there, our hearts ache.

### CASUAL WARDS IN LONDON: SUPERINTENDENTS, ACCOMMODATION, AND SITUATION.

Statement Showing the Parishes and Unions in the Metropolis in which there are Casual Wards, the Situation, and Number of Separate Berths in Each, and the Names of the Superintendents.

| District. | Union or Parish and Situation of Casual Wards. | No. of Separate Berths. | | | | Names of Superintendents of Casual Wards. |
|---|---|---|---|---|---|---|
| | | Males. | Females. | Females with Infants. | Total. | |
| West | Fulham, Margravine Road | 42 | 20 | 5 | 67 | Mr. C. Greyson. |
| | Kensington, Mary Place, Notting Hill | 40 | 12 | 8 | 60 | Mr. G. Perry. |
| | Paddington, Harrow Road | 46 | 24 | — | 70 | Mr. H. Hooks. |
| | St. George's, Wallis Yard, Buckingham Palace Road | 38 | 19 | 1 | 58 | Mr. F. Godfrey (attendant on Male Casuals). |
| North | Hackney, Workhouse, High Street, Homerton | 26 | 10 | 6 | 42 | Mr. T. Page. |
| | Hampstead, New End | 10 | 9 | — | 19 | Mr. C. Weekley. |
| | Islington, St. John's Road | 46 | 24 | 6 | 76 | Mr. B. Nancarrow. |
| | St. Marylebone, David Street, Baker Street | 65 | 26 | 3 | 94 | Mr. C. Skinner. |
| | St. Pancras, Bower Cottage, Leighton Road | 34 | 28 | 4 | 66 | Mr. W. R. Stuart. |
| Central | City of London, Robinhood Court, Shoe Lane | 54 | 36 | — | 90 | Mr. W. Tucker (Superintendent). Mr. G. M. Humlington (Night Porter). |
| | Holborn, Vine Street, Clerkenwell Road | 49 | 30 | — | 79 | Mr. G. A. Colton. |
| | St. Giles and St. George, Bloomsbury, Macklin Street | 52 | 19 | 5 | 76 | Mr. W. Duffus. |
| East | Mile End Old Town, Bancroft Road | 22 | 4 | 6 | 32 | Mr. G. W. Franklin. |
| | Shoreditch, Nuttall Street, Kingsland Road | 30 | 9 | 4 | 43 | Mr. C. Hatcher. |
| | St. George-in-the-East, Old Gravel Lane | 50 | 18 | — | 68 | Mr. J. Matthews. |
| | Whitechapel, Thomas Street | 44 | 25 | — | 69 | Mr. A. Edmonds. |
| South | Camberwell, Nazareth House, Gordon Road, Peckham | 39 | 16 | 8 | 63 | Mr. J. Williams. |
| | Greenwich, Woolwich Road | 40 | 10 | 6 | 56 | Mr. W. Stephens. |
| | Lambeth, Lucretia Street | 40 | 32 | 6 | 78 | (Vacant.) Mr. E. Fitt (Assistant). |
| | Lewisham, High Road | 30 | 16 | 8 | 54 | Mr. H. Parker. |
| | St. Olave's, Rotherhithe | 40 | 8 | 4 | 52 | Mr. R. Palmer. |
| | St. Saviour's, Mint Street (for Males Only) | 30 | — | — | 30 | Mr. J. R. Williams. |
| | St. Saviour's, Westmoreland Road, Walworth (for Females Only) | — | 30 | — | 30 | Elizabeth Reynolds (Wardswoman). |
| | Wandsworth and Clapham, St. John's Hill | 69 | 44 | 4 | 117 | Mr. G. Cheshire. |
| | Woolwich, Plumstead | 20 | 5 | 5 | 30 | Mr. J. Beavan. |
| | | 956 | 474 | 89 | 1519 | |

### THE PRESENT SYSTEM OF OUT-DOOR RELIEF.

Granted that certain persons must of necessity become paupers, and that many of them will become such through no fault of their own; and granted that the community is fully agreed that it is right and proper they should be relieved; it is desirable that deserving cases shall be relieved with as much regard to their own comfort and convenience as may be possible. For it must never be forgotten that the poor are a large number, and include persons of every variety of education and moral character; and to treat all these as if they were a mere herd of unredeemed idlers and loafers is as stupid as it is inhuman. The question of out-relief for the poor is one requiring for its general management the wisest instructions, and for its detailed administration the nicest discrimination. It is admitted that the workhouse test has operated powerfully in the diminution of the number of out-door paupers; and to a very large extent that diminution is an unmixed good. Those who in small towns and villages have had opportunities of becoming familiar with the character and habits of paupers, know that there are certain individuals who will always be on the rates if they can be so without the loss of their liberty. There are hereditary paupers as there are hereditary peers, and the writer remembers cases where grandparents, parents, grandchildren, and great grandchildren have all been chargeable to the same union at the same time. In such families a more self-respecting and independent individual may now and again appear; but the rule will be that the family, as a family, will, in all its generations, be in a condition of chronic pauperism. In these cases the house test is invaluable, and should be rigorously applied. It is probable that in populous towns a still larger proportion of hereditary and easily-produced paupers have to be reckoned upon, because in the lower social strata of such towns the public opinion which in a small country town operates over the whole population and maintains among the poor a considerable degree of what may be called "decent shame," is comparatively powerless. The poor are separated by an almost impassable gulf from the better classes, and being left to themselves rapidly deteriorate in regard to the higher social qualities. With such a class the application of the house-test should be strict and very general; and here, as in the country, its employment has, as a matter of experience, been followed by a large diminution in the number of paupers, and in the amount spent on their relief.

### Worthy of Intelligent Consideration.

So far well! But it is important to bear in mind that in the metropolis, although the number of paupers has been largely diminished by the house test, the relative amount annually expended on poor relief is now, in proportion to the whole population, larger than it was before this test was so generally and vigorously applied. As we show elsewhere, it cost nearly £600,000 more to relieve 101,443 paupers in 1886 than it cost to relieve 115,010 in 1874. Besides, if we ourselves were in the position of involuntary and most-ashamed-of-ourselves paupers, it is probable that we should consider a little modification of rules highly desirable, and some elasticity of administration in every way a thing to be approved. Can it be either wise or humane to rank all paupers together as persons against whom society must always be in an attitude of defence? Are there not, and must there not always be, a number of thoroughly well-conducted persons who, as they become older and feebler, will insensibly graduate downwards until the dreadful region of pauperism is reached? And are these, then, to be pitched into, and for evermore remain in the degrading cauldron of half-thieves, half-drunkards, incapable fools, and hopeless reprobates, who constitute a large proportion of the pauper army? If they are, and we are to acquiesce in such a doom for them, some of us will at least have the grace to blush for ourselves and our fellow-countrymen. We know now how valuable the house-test is, and the administrators of the law have acquired much skill in its use. Is it not time to relax its stringency in the case of the aged, or those who have been disabled by illness, to such an extent at any rate as to allow them to maintain themselves in part by their own exertions? All that is required is a sufficient degree of intelligence and humanity on the part of the relieving officer to prevent any abuse of the modification. Many boards of guardians in large unions have succeeded in reducing the out-relief largely by exchanging districts, so that no board shall deal with individuals known to its own members. That is no doubt result on which they may congratulate themselves rom a ratepayer's standpoint. But there is still the pauper's view of the question to be considered; and surely none of us need be ashamed to confess that we think that the decent pauper is entitled to a little consideration. We profess to be a Christian nation, let us never forget that no amount of poverty can shut a man out of that community which claims "all sorts and conditions of men" for its fellowship. We ought to admit to ourselves, and so far as we can, enforce by means of those whom we employ to administer the rates, this general principle of humanity and religion: "Whatsoever ye would that men should do unto you, do ye even so unto them." Nobody surely will say that the value of the principle is impaired because it is recognised and enforced in the New Testament.

### Relieving Officers and Workhouses.

The name and address of every relieving officer, and master of a workhouse, arranged in districts, will be found upon pages xiii., xiv., xv. No hours of attendance are given because master or matron must always be on duty at the workhouses, and relieving officers must be available at all hours. At least, this is the theory of the present system, and, like many others, it is found in practice to be neither satisfactory nor fair to the officials or to those who need their aid and counsel.

### WHAT REMAINS TO BE DONE.

### General Considerations.

Having now completed our account of the charitable and State agencies in London, which minister to the needs of the poorer population, we have next to consider what remains to be done. It will be seen that, assuming one of the unemployed or casual poor is in a position to know to what agencies he can apply for assistance in his distress, the State agencies, at any rate, are so conducted, and so limited, as to make it well nigh impossible for the self-respecting members of this class to apply to them for aid at all. This is a very serious aspect of the present course, which admits, and will no doubt receive, the careful consideration of all who bring their minds to bear upon the question of the day. Unfortunately, though we may recognise the evils and shortcomings of the system, we cannot remedy it in a moment, and so practical people must proceed to examine the wider question of how to face the needs of the unemployed and casual poor within the metropolitan area, in order to realise justly and exactly the extent of those needs, and to meet them adequately and well. These considerations make it necessary to recall for the moment the general impression which prevails on the subject, both amongst Londoners and the world at large.

### London as a Whole.

The problem here presented is so vast that it terrifies and repels many minds, and so cripples their energies. Thus, Mr. Robert T. Paine, jun., the President of the Associated Charities of Boston, writes: "Cities of the size and needs of London and New York are alone too vast for our complete grasp. In every other city of America and of Great Britain it must be possible in no long time to organise a corps of visitors, who, in the ways now developed, can find or furnish at least one friend for every family in need. Miss Octavia Hill, who knows the metropolis well, says of London in regard to this question, "the problem has become appallingly gigantic. Viewed in its entirety, it might almost make us tremble." Viewed in its entirety—that is the keynote to the position, for, as the largest building is but an aggregation of bricks, stones, and mortar, so a vast city like London is but an aggregation of streets and houses and small communities, which, if taken in detail, can be dealt with as easily as any other town or city, wherever it may be located. Now, the Redistribution Bill did a great service for London, if the inhabitants will only recognise and act up to the opportunities which it has given. London consists now of thirty-seven Parliamentary constituencies or divisions, and if each division will take up the question of the unemployed and distressed within its boundaries, and if the leading inhabitants will associate themselves together for the purposes of ascertaining by degrees, but systematically, the circumstances, condition, and needs of every poor family in their district, then the problem will be capable of easy solution. The failure of all previous efforts to effectually grapple with the uestion up to the present is due entirely to the fact that a great central fund like the Mansion House Fund has been raised and distributed without adequate knowledge, and so failure and disappointment have necessarily resulted.

### The Difficulty of To-day.

Yes, absence of knowledge is the difficulty of the moment. We have no compass or chart to tell us where we are or on what course we ought to steer. Until both these omissions are supplied it is impossible for anybody to act with wisdom in this matter, and they had therefore better pause before they act at all. In truth, though we are desirous that an abundance of money should be forthcoming to meet the legitimate claims and necessities of every poor family in our midst, we believe that no fund ought to be raised until an accurate knowledge of all the circumstances has been obtained. No one who understands the bearings of London's great problem can deny that the raising of Mansion House funds on the basis of past efforts of this description tends to increase the difficulty immeasurably rather than to supply the wants of the really deserving poor. Having no knowledge of the right kind, every Parliamentary district throughout London should proceed to obtain it, each for itself. Our contemporary, the *Pall Mall Gazette*, is urging the Members for each metropolitan division to undertake to open a register in which the unemployed may enter their names. There can be no doubt that registration will ultimately prove to be the compass which will take us into port; but our knowledge of the systems of registration in force, which have proved successful in other countries and places, compels us to say that the proposals of the *Pall Mall Gazette* will not meet the case. It is with regret that a unique experience compels us to make this statement, but we are convinced that our con temporary will be the first to admit its justice when we proceed to explain.

### What Registration Means.

Registration rightly understood and applied, must produce accurate information concerning the actual necessities and real position of each of the families whose names are thereby recorded. To secure this it is essential to have not only a register of unemployed, but a register containing a full and true record of every family receiving relief. We therefore feel, as the Boston experience has shown, that the first thing to be done is to establish a clearing-house of all relief given by all agencies and persons, and of all information collected by them. Any registration office to prove successful must do work of two kinds—it must gather and give information. First of all, it must collect reports of all relief, all information as to the circumstances of each family or person registered; secondly, and this is even more important than the first, for it gives to it its chief value, it must so arrange its information as to be able to give a prompt reply to any inquiry, and must be able to state at once who is relieving a particular case, and what relief is being given. Each society and individual before relieving a person is entitled to know what relief he gets from other sources. Sometimes too much relief may be given, and sometimes too little. The essence of the plan is to secure exact knowledge of the facts, and so to add to the fitness of the gift made or work found, as the case may be. It is, therefore, not only necessary to open registration offices where the unemployed can record their names, but it is also necessary to provide that by registering the cases receiving relief, every relief agency, whether poor-law or voluntary, every church and every charitable person is called upon to help, not only to work for the poor, but to work in co-operation, and so to become parts of the great organisation which shall ultimately result in the poor of London, their difficulties, needs, and trials, being as wel understood and as thoroughly—nay, more thoroughly—provided for than in any town or community that may be named.

### A Hint to Metropolitan Members.

Lord Randolph Churchill has taken the right course in Paddington in regard to this question. He recognises that it is a discredit and a reflection upon all classes in London that the present ignorance and unmethodical treatment of the poor should continue. He is impressed with the feeling that London's great problem is worthy of the combined, cordial, and earnest efforts of all parties and of all creeds, and that it would be little short of disgraceful to allow it to degenerate into a question of party, of dogma, or of class. He has caused the representative residents of every shade of opinion and every creed to be invited to meet him in conference, with the result that a committee has been appointed to consider and report how the chronic distress can be continuously dealt with and provided for, not during the present winter but for all time, so far as Paddington is concerned. With the desire of removing all party complexion, Lord Randolph has thought it best to refrain from joining the committee, of which the Vicar of Paddington is chairman. Why should not every Metropolitan Member adopt a similar course by inviting the representative men and women of all classes to meet him in conference, with the view to ascertaining how best to deal with this question, so far as their district is concerned? Individually the London resident can do very little, but collectively, and on this system, much may be done, for by this means a wise and final solution may be found.

### When the Facts are Known.

Reverting once more to the registration of the unemployed, and of all classes in receipt of relief either from the poor law or from charities, we have now to consider what can be done when the facts are ascertained. Every district which systematically establishes and works the registration system on the lines here laid down, will gradually come to know exactly what are the actual needs of the poorer population within its limits. We shall no longer have to deal with impressions, and for this reason it will no longer be possible for anybody to sneer at the kindly efforts of the noble-hearted philanthropists who have established and worked Mansion House and other funds in the past. When the facts are known, as they will be if registration is generally adopted, then it will also be possible to ascertain definitely to what extent, if at all, a charitable fund is desirable, and the amount which must be raised for it. There will then be no more failures, because every penny of the money will be paid out for a definite purpose. Each family or individual receiving a grant will do so because it has been ascertained that it is not only deserved, but necessary to provide clothing, tools, shelter, and other definite needs, with the object of lifting the recipients to a position, where by honest effort they may maintain themselves in respectability and independence if not in comfort. In short, every poor and worthy person resident in London may in time be provided with a friend, and so encouraged to hope for and ultimately to secure a prospect for those dependant upon him. If this can be done, as it certainly may be, on the system here advocated, what man or woman amongst us is there who will not thank God and take courage?

### Work for the Unemployed.

We can well believe that many who have read to this point have impatiently said to themselves: "This is all very well, but how about the unemployed? Are they to be provided with work if they are willing to undertake it?" This feeling, though natural, is not quite practical, and we would remind all such readers of the results of the census recently taken by the Government at the request of the

Socialists. This census has been admitted on all hands to be largely valueless, and why? It contains only such statements as the individuals met with have consented to make, and it is, therefore, one-sided; and so believed to be largely unreliable. So in the matter of the unemployed. Experience has shown that the men who are loudest and most earnest in their demands for work subsequently prove to be unwilling to undertake it when work is found for them. Hence the necessity for caution and knowledge at the outset, and for this reason we have put registration before work for the unemployed. Earnest men who contend that it is possible to provide work for the unemployed, are at once met with certain objections which seem at first to be insurmountable. It is urged—(1) That artisans and skilled mechanics, watchmakers, finishers, bootmakers, tailors, and others, cannot be provided with work upon improvements, or indeed at all outside their own proper trades; (2) that it is impossible to find work in London for the unemployed which shall be genuine, as opposed to task work; (3) that, assuming work is found, it will be impossible to provide funds to carry out public works to an extent sufficient to provide for the needs of the unemployed. Let us consider each of these objections in detail.

(1) Can Artisans be Usefully Employed as Navvies?

The only way to answer this question is to ascertain the teachings of experience on the subject. The public works established in connexion with the Lancashire Cotton Famine, when £1,850,000 was provided by Parliament to secure work for the unemployed in the manufacturing districts of Lancashire affords a case in point. Everybody should read at this time the report of Sir Robert Rawlinson, C.B., the Government Engineer to the Local Government Board, published in the twenty-first annual report of the Poor Law Board, 1868-69, pages 105 to 153. He says: National public works have been established in modern times for several purposes—as to find employment for workmen in times of great stagnation of trade and local distress, or to carry out public works, such as new roads and various forms of earth works. Objections were taken in Lancashire to the proposed scheme of employing the distressed men on works because a time of local distress was not favourable to commencing works by unskilled men, to be paid for out of the local rates; because it was considered by many of the large manufacturers that these unoccupied men could not with any advantage execute such works, and that those who did work with pick, spade, or barrow, would have their hands and fingers ruined, and their constitutions broken down under exposure; that necessarily a large proportion of the money required would have to be paid to skilled labourers other than distressed cotton operatives, and also that very large sums of money would have to be expended on plant and materials. The reply was: The able-bodied man out of work must either be maintained out of the poor rates or by charity, or by rates and charity combined, and the continuance of such relief would utterly demoralise the men. I asserted that I knew by former experience that the best of the distressed cotton operatives both could and would soon learn to do a fair day's work for reasonable day's wages, if paid in money and at shor intervals. The distressed men employed had been overlookers, weavers, spinners, card-room hands, workers, piecers, twisters, sizers, dyers and bleachers, fustian cutters, roller coverers, hatters, and members of various other trades; the weekly wages, working either by day's wages or to measure and value, varying from 12*s.* to 20*s.* per man. As a rule each man so employed represented a wife and three children, or five in a family.

Sir Robert Rawlinson's Experience.

After describing the works executed, and pointing out that drainage for sewers and other forms of work undertaken required the combined action of skilled men to open and temper the drainage, but unskilled men to bring materials to the site, and remove surplus earth, Sir Robert proceeds to give the results of the experiment so far as the men were concerned, as follows:—At first the pick, the shovel, and the barrow galled the hands of men only accustomed to handle cotton thread, but the encouragement of the local authorities and local relief committees in offering a subsidy to each man going to work for a space of six weeks, and the discriminating assistance of the Central Relief Committee—with the Earl of Derby at its head—in granting useful clothing to several thousand men, and waterproof boots to those who were required to work in wet ground, encouraged the men to persevere. In a short time the men's hands hardened, whilst fresh air and exercise brought them into condition, enabling them to feel at ease instead of suffering stiffness and pain. Many of the men in three months' time increased in strength, and became comparatively skilled workmen, capable of executing sewers, drainage, and other forms of earth work requiring both skill and care. Sir Robert declared that up to the present time he is not aware of any failure in the works undertaken. Thus the first objection to providing work for the unemployed is proved to be untenable when tested by experiment. It shows that men whose previous occupations had been indoors learnt forms of out-door labour very rapidly; but this was principally because there was sub-division, and because the distressed men received proper encouragement, and, above all, technical employment. If the distressed cotton operatives had merely been offered warm clothing, employment, and labour, to work in large gangs, the experiment would, in Sir Robert's opinion, have failed, as complainers and grumblers would have leavened such gangs, and I believe would have constituted themselves leaders.

The provision of this work relieved the local rates by the removal from the local relief lists of numbers of men who would not work, and would otherwise have received a dole of 3*s.* or 4*s.* a-week, and lived in idleness to the end of the distress. Besides, the provision of public works for the unemployed is twice blessed, it blesses those who work and those who provide the work. In Lancashire the large towns have benefited trade by substituting 400 miles of good road for tracts of mud. They have further added to local means of health and pleasure by providing public parks and recreation-grounds, which otherwise might not have been formed, and have increased the rental value of house property by sewering and draining, and so removing nuisances from the vicinity of dwelling-houses, which nuisances, if allowed to remain, would have been liable to injure the public health. This evidence, as we have said, removes objection No. 1.

[2] Cannot Suitable Public Works be Found in London?

This objection falls to the ground when examined closely. We have ascertained from the highest living authority on the subject, whose name we are not at liberty to mention, that an abundance of public works of a suitable character may be found in London for the unemployed, if the inhabitants have only the will to insist upon this being done. To be successful, the following points must, however, be insisted upon: In recommending advances of public money for the employment of able-bodied men out of work, we must so arrange the central administration that it shall not divest the Local Authorities of their responsibility. The work offered to the men must not in any case be test or task work, but work for wages at prices fixed beforehand with the men, so that the payment made to each man, or to each gang of men, shall depend upon the actual measured amount of work done each day, week, or month, as the case may be. In Lancashire the works were to be of public utility, such as (1) town sewering and house draining; (2) storage reservoirs for water supply; (3) improving streets and roads; (4) forming public parks, recreation-grounds or public cemeteries, &c.;

(5) forming public markets; (6) land drainage and improving waste lands; (7) river improvements. We have not space to-day to dwell in detail upon the many works of public utility which might be undertaken in the various Parliamentary divisions of the metropolis. We shall content ourselves with pointing out that there are several waste lands within easy distance of the metropolis which might with advantage be drained and improved, Barnes Common being an example. The Corporation of London might no doubt, as on former occasion, provide employment for a number of men upon works of public utility in connexion with Epping Forest. Such schemes as these would, however, be of a temporary character, and if we knew the facts they might prove—though we much doubt this—too small to provide employment for the whole of the unemployed of London who would prove willing to work at the present time.

Be this as it may, if London really arouses itself to its responsibilities in this matter, these works must prove inadequate to provide employment for some years for such cases as we have described, and we have therefore devoted much time and thought to the search for means whereby suitable work may be secured for all the unemployed of London during the next ten years. We have the very highest authority for declaring that we believe this work will be found in connexion with the purification of the River Thames and the disposal of the sewage of London. A Royal Commission, over which Lord Bramwell presided, was appointed (1) to inquire whether any evil effects resulted from the system of sewage discharge into the Thames, and (2) in that case what remedies could be applied. After hearing counsel and ample evidence during the latter half of 1882 and the greater part of the year following, the Commissioners reported in January, 1884, on the first branch of their inquiry. One weak point in the system of (metropolitan) main drainage works was the storm overflows which allowed occasional discharges into the river of considerable quantities of offensive matter. Great objection was also made as to the discharge of crude sewage, and reference was made to the state of the Thames at the outfalls. Before their second and final report, dated November, 1884, the Commissioners took evidence upon proposals to remove the outfalls lower down the river or to the sea; the expediency of separating sewage from rainfall; the utilisation of metropolitan sewage, with the prospect of doing so at a profit, and various methods of treating sewage and obtaining a pure effluent, as by broad irrigation, filtration through land, and deposit or precipitation. They next expressed a stronger opinion than in their first report on the evils resulting from the outpour of sewage into the Thames, especially in a crude state, as "a system neither necessary nor justifiable." After dealing with various means for lessening the evils of the existing system, they declared, if suitable land could not be procured near the existing outfalls any liquid residue should be conveyed lower down the river. The main conduit might then, if thought desirable, be made of sufficient capacity to include a general extension of the drainage to all the districts around London, as recommended by Sir Joseph Bazalgette and Mr. Baldwin Latham. In the new drainage works the sewage should be, as far as possible, separated from the rainfall.

Anyone, and especially any member of the press, who wishes to guide Londoners to a right conclusion in this matter, should refer to the reports and evidence of the Royal Commission, and also to a report of Sir Robert Rawlinson, C.B., on an inquiry upon the condition of the Thames at the Barking outfall, issued in 1869. They will find in the latter report a valuable map, showing the various routes the main conduit might take, and it is evident from this report, though issued long prior to that of the Royal Commission, that Sir Robert is distinctly favourable to the proposal. Of course—we say of course because no reasonable person can expect much from this authority after the recent revelations and its action thereon—the Metropolitan Board of Works have not adopted the recommendation of the Royal Commission, and declare it to be impracticable, although some of the most eminent engineers are, it is stated, prepared to undertake the work, and to carry it through to a successful issue. Londoners who are proud of their river resent its present condition, which Mr. Thornhill Harrison (see Parliamentary paper No. 323, of 1884) has thus described: "The Thames in its present condition can only be compared to a huge sewer tank, which for many months has not been cleaned out." This huge sewer tank is the work, and, if we are to judge by their action, it would seem to be the pride and glory of the Metropolitan Board of Works. Will Londoners permit this authority to pollute and foul their river any longer? Until to-day this question has stood alone, but it now involves the further question as to whether or not London is to provide works of public utility for the unemployed to-day. We have it on eminent authority that if the recommendations of the Royal Commission be carried out, the construction of the main conduit or conduits will provide employment, if necessary, for 10,000 men; and further, that the works, which will necessarily occupy several years in construction, if and when completed, may still be utilised, if irrigation follow under a proper system, for the purpose of giving employment to a relatively few or a relatively large number of the unemployed of London, as circumstances may from time to time require. We can then at one and the same time purify the Thames and provide work for the unemployed if we wish to do so—an important fact, and one worthy of the serious attention of all.

### How Funds are to be Provided.

This brings us to the third and last objection, that when the work has been found, funds cannot be provided to pay for it. London is so badly governed at the present time, that the various districts are placed at a disadvantage as compared with municipalities and local authorities in the provinces. Still the metropolis can, if adequate pressure is brought to bear upon it by the inhabitants of any district, acting through its vestry, provide money, by laying in sufficient amounts to enable works of public utility in a particular district, to be commenced without great delay. The Metropolitan Board of Works exercise their borrowing powers subject to the sanction of the Treasury. In 1885 Parliament allowed the Board, with the consent of the Treasury under certain conditions, to raise money required for temporary purposes, by means of bills somewhat in the form of Treasury bills. Such bills are charged on the Metropolitan consolidated rate with a condition for repayment in not less than three or more than twelve months after date. A Money bill is passed every Session empowering the Metropolitan Board to borrow the money which will be required for various purposes during the succeeding year. If the inhabitants choose to move the vestries to move the Board of Works, and if the Treasury—*i.e.*, the Government is willing to co-operate—as we believe it is—to provide employment upon any reasonable basis, there will be no technical or local difficulty to prevent them from doing so. Of course the larger question of immediately commencing the main conduit referred to above may require a special Act. We are, however, advised on high authority that if the Government will provide £500,000 there is sound reason for believing that this work—if undertaken and carried out with equal care and judgment to that displayed in the manufacturing districts in 1862-67—may be executed with equal success, socially, financially, and morally. We have already shown the social and moral success of the experiments in Lancashire, and the financial success will be recognised when we state that of the whole sum of nearly £2,000,000 advanced by the Public Loan Commissioners, all has been repaid with the exception of £472,813.

THE END OF THE WHOLE MATTER.

The above statements are made on the authority of official and published documents, and after consultation with eminent authorities in their several branches of work, and it may be taken for granted, therefore, that the facts are as stated. It rests entirely with the people of London and the metropolitan press to determine whether they will proceed to solve London's [illegible]blem once and for all. It must be admitted by all reasonable persons that the solution rests entirely with the people themselves, assisted by the press. The result will not be arrived at without much sacrifice and devoted labour on the part of individuals; but the object to be attained is of such gigantic importance, and so noble in itself, that we believe the humanising influence of the bare thought of its possibility will extend and circulate through every district of London; and that each district will declare through its principal inhabitants, at the call of its Parliamentary representative, its determination to do its full proportion zealously and well. In such circumstances, instead of death by starvation and harrowing scenes of misery and want, we, as residents in this vast metropolis, shall know for certain that, wherever any family has fallen so low as to need relief, we shall be enabled to send to them at least one friend, to do for them all that a friend can do to discover and remove the cause of their dependence, and to help them up into self-support and self-respect. Pauperism will be reduced to the smallest possible limits, for the Lancashire experiment proved that the prevention of pauperism by means of public works was at least to the extent of three times the number of men employed upon them. If Londoners will, that is the end of the whole matter. What will be the answer of the public and the press? We must leave the public and the press to supply their own answer.

---

### THE RELIEVING OFFICERS THROUGHOUT LONDON.

**Names, Districts, and Addresses.**

| Name of Union and No. of District. | Relieving Officer and Address. |
|---|---|
| **Bethnal Green,** No. 1 | Mr. W. H. Dormer, 78 Gore Road. |
| No. 2 | Mr. R. Arnott, 10 St. Peter Street. |
| No. 3 | Mr. C. A. Forrest, 216 Wilmot St. |
| No. 4 | Mr. W. A. Bowak, 20 Durant St. |
| Workhouse Master— | *Mr. R. L. Sturtevant*, Bonner's Hall Field, Bethnal Green, E. |
| **Camberwell,** No. 1 ... | Mr. J. Comfort, 135 Gordon Road, Peckham. |
| No. 2 ... | Mr. J. Sedgley, 117 Bushey Hill Road, Camberwell. |
| No. 3 ... | Mr. C. Followes, 135 Wells Street, Camberwell. |
| No. 4 ... | R. Purkis, 47 Hill Street, Peckham. |
| Workhouse Masters— | *Mr. F. Wint*, Havill Street Workhouse, Camberwell, S.E. |
| | *Mr. J. Stevenson*, Gordon Road Workhouse, Peckham, S.E. |
| **Chelsea,** North-West | Mr. F. G. Crouchey, 7 Caversham Street (at present, but is going to remove shortly). |
| North-East | Mr. G. W. Faryon, 264 King's Road, Chelsea. |
| South ...... | Mr. W. G. Holland, 8 Glebe Place, Chelsea. |
| KensalTwn. | Mr. J. W. Facey, 163 Kensal Road. |
| Workhouse Master— | *Mr. C. Wright*, Arthur Street, St. Luke, Chelsea, S.W. |
| **Fulham,** No. 1 ......... | Mr. H. E. Rogers, 29 Horder Road, Fulham. |
| No. 2 ......... | Mr. W. Wood, 33 Aspenlea Road, Fulham. |
| No. 3 ......... | Mr. W. F. Halsey, 11 St. Peter's Road, Hammersmith. |
| Workhouse Master— | *Mr. G. F. Knott*, Fulham Palace Road, Hammersmith, W. |
| **St. George's in the East,** No. 1 (South) | Mr. J. Barnes, 222 Cable Street. |
| No. 2 (North) | Mr. G. Merritt, 282 Commercial Road, E. |
| Workhouse Master— | *Mr. J. Tyler*, Princes Street, Old Gravel Lane, E. |
| **St. George's,** A......... | Mr. J. Wray, 2 Montpelier Terrace, S.W. |
| B......... | Mr. J. West, 46 Westbourne Street, S.W. |
| C......... | Mr. W. A. Madeley, 26 Colchester Street, S.W. |
| D and E | Mr. W. Collis, Superintendent, 118 Vauxhall Bridge Road, S.W. |
| D......... | Mr. E. J. Badderly, 100 Horseferry Road, S.W. |
| E......... | Mr. R. B. Hannant, 67 Horseferry Road, S.W. |
| Workhouse Masters— | *Mr. F. E. Elkington*, Wallis Yard, Buckingham Palace Road. |
| | *Mr. Geo. Cole*, Fulham Road Workhouse. |

| Name of Union and No. of District. | Relieving Officer and Address. |
|---|---|
| **St. Giles - in - the Fields and St. George, Bloomsbury** (the Parishes) | Mr. R. J. Goldspink, 9, Compton Street, Brunswick Square, W.C. |
| Workhouse Master— | *Mr. C. R. Ellis*, Endell Street, W.C. |
| **Greenwich—** | |
| Deptford (North) | Mr. B. Dark, Mary Ann's Buildings, Deptford. |
| Deptford (South) | Mr. W. Pilcher, Watson Street, Deptford. |
| Deptford (West)... | Mr. W. T. Street, Simla Street, Deptford. |
| Greenwich (East) | Mr. W. H. Peters, Norfolk Place, Greenwich. |
| Greenwich (West) | Mr. W. Wates, Royal Hill, Greenwich. |
| Workhouse Master— | *Mr. W. H. Jordan*, Greenwich, S.E. |
| **Hackney,** No. 1 ...... | Mr. E. L. Crane, 83 Olinda Road, Stamford Hill, N. |
| No. 2 ...... | Mr. J. Buckmaster, 49 Almack Road, Lower Clapton, E. |
| No. 3 ...... | Mr. T. Draper, Relief Offices, Dalston |
| No. 4 ...... | Mr. J. B. Frost, 29 Chatham Place, Hackney. |
| No. 5 ...... | Mr. J. Hallett, 37 Christie Road, South Hackney, E. |
| No. 6 ...... | Mr. W. Pease, 38 Harcombe Road, Stoke Newington, N. |
| No. 7 ...... | Mr. E. P. Fenton, 60 Malvern Road, Dalston, E. |
| No. 8 ...... | Mr. C. Jarvis, 127, Bentham Road, South Hackney. |
| No. 9 ...... | Mr. J. R. Marriott, 92 Clifden Road, Clapton Park. |
| Workhouse Master— | *Mr. J. Mason*, St. John, Hackney, E.* |
| **Hampstead** Parish... | Mr. C. Weekley, New End, Hampstead. |
| Workhouse Master— | *Mr. J. H. Forbes*, Hampstead, N.W. |
| **Holborn—** | |
| Clerkenwell (Sothrn.) | Mr. E. Barber, 49 Baker Street, Clerkenwell. |
| Clerkenwell (Nrthrn.) | Mr. F. Strickland, 42 Cumming St. |
| Clerkenwell (Central) | Mr. A. G. Pratt, 32 Wilmington Sq. |
| St. Luke's (Eastern) | Mr. G. R. Trappes, 318 City Road. |
| St. Luke's (Western) | Mr. E. R. Smith, 33 President Street. |
| St. Luke's (Northern) | Mr. W. H. Toynbee, 304 City Road. |
| Holborn (Eastern) ... | Mr. G. Goldsmith, 16 Wharton Street, Clerkenwell. |
| Holborn (Western) ... | Mr. F. H. Birch, 12 Millman Street, Bedford Row. |
| Workhouse Masters— | *Mr. G. Merryweather*, 158 Gray's Inn Road. |
| | *Mr. W. Daniells*, Shepherdess Walk, City Road, E.C. |
| | *Mr. W. Cuthbe*, Mitcham. |

* In the *London Directory* it is given as "Sidney Road, Homerton, E."

| Name of Union and No. of District. | Relieving Officer and Address. |
|---|---|
| **Islington,** No. 1 ...... | Mr. R. G. Thomas, 66 Mulkern Road. |
| No. 2 ...... | Mr. S. O. Cambridge, 45 St. John's Road, N. |
| No. 3 ...... | Mr. A. T. Peacey, 3 Hartham Ter. N. |
| No. 4 ...... | Mr. J. J. Agar, Relief Offices, Highbury Mews. |
| No. 5 ...... | Mr. T. Phillips, 107 Mildmay Road. |
| No. 6 ...... | Mr. A. H. Dickinson, 22 Thornhill Crescent. |
| No. 7 ...... | Mr. R. S. Merrifield, Relief Offices, Liverpool Road. |
| No. 8 ...... | Mr. G. Craig, 1 Sebbon Street. |
| No. 9 ...... | Mr. J. Scannell, 3 Cannon Street, New North Road. |
| Workhouse Master— | *Mr. G. Sustins*, St. John's Road, Upper Holloway. |
| **Kensington,** No. 1 ... | Mr. W. B. Neville, 65 Chesterton Road, Notting Hill. |
| No. 2 ... | Mr. F. Vassie, 149 Chesterton Road, Notting Hill. |
| No. 3 ... | Mr. F. C. Coombes, 17 Princes Road, Notting Hill. |
| No. 4 ... | Mr. E. Hickox, 84 Abingdon Road, Kensington. |
| No. 5 ... | Mr. H. Essex, 3 Hollywood Road, West Brompton. |
| Workhouse Masters— | *Mr. G. Aylward*, Marloes Road, Kensington, W. |
| | *Major E. Dillon*, Mary Place, Notting Hill, W. |
| **Lambeth,** No. 1 ...... | Mr. E. Singleton, 11 Manners Street, York Road, S.E. |
| No. 2 ...... | Mr. S. Ames, 126 Kennington Road, S.E. |
| No. 3 ...... | Mr. B. C. Parker, 133 Lower Kennington Lane, S.E. |
| No. 4 ...... | Mr. T. K. Baldock, 18 Trigon Terrace, Clapham Road, S.W. |
| No. 5 ...... | Mr. J. T. Lowing, 367 Kennington Road, S.E. |
| No. 6 ...... | Mr. H. J. Cuming, 29 Lansdowne Road, S.W. |
| No. 7 ...... | Mr. J. H. Barker, 117 Loughboro' Road, S.E. |
| No. 8 ...... | Mr. J. T. Hearson, 2 Zingari Villas, Lower Norwood, S.E. |
| No. 9 ...... | Mr. J. F. Eastey, 2 Vauxhall Street, S.E. |
| Workhouse Masters— | *Mr. F. Fielder*, New Workhouse, Renfrew Road, Lower Kennington Lane, S.E. |
| | *Mr. R. Clinton*, Old Workhouse, Princes Road, S.E. |
| **Lewisham—** | |
| No. 1 (Lewisham) | Mr. J. N. Mott, Relief Station, High Street, Lewisham |
| No. 2 (Lee and Eltham)............ | Mr. T. D. Higgins, Relief Station, Lee Green, Lee. |
| No. 3 (Sydenham and Forest Hill) | Mr. W. C. Rich, Relief Station, Waldram Road, Forest Hill. |
| Workhouse Master— | *Mr. J. Franklin*, High Street, Lewisham, S.E. |
| **London, City of—** | |
| No. 1A...... | Mr. J. C. Webb, 16 Bouverie Street, Fleet Street. |
| No. 1B...... | Mr. A. Brown, 1 Hayne Street, Charterhouse Square. |
| No. 2 ...... | Mr. S. Alloway, 93 Bishopsgate Street Without. |
| No. 3E...... | Mr. W. Bosher, 103 Bishopsgate Street Without. |
| No. 3F...... | Mr. T. A. Chapman, 8 Sparrow Corner, Minories. |
| Workhouse Master— | *Mr. H. Rambsbottom*, Homerton, E. |
| **St. Marylebone—** | |
| St. John's ............... | Mr. W. Parsons, Charles Street, Portland Town. |
| Christchurch (A) ...... | Mr. C. J. Hunt, Little Union Place, Lisson Grove. |
| Christchurch (B) ...... | Mr. G. C. Soper, 17 Stafford Street, Marylebone Road. |
| St. Mary's............... | Mr. J. W. Swyer, 15 Harcourt Street, Marylebone Road. |

| Name of Union and No. of District. | Relieving Officer and Address. |
|---|---|
| ST. MARYLEBONE—(*continued*). | |
| Rectory ................... | Mr. W. T. Cooper, 47 East St. N.W. |
| All Souls & Cavendish Square ............... | Mr. H. J. Stranack, 18 Charlotte Street, Portland Place, W. |
| Workhouse Master— | *Mr. G. E. Douglass*, Northumberland Street, W. |
| **Mile End Old Town—** | |
| (Eastern) | Mr. J. B. Slight, 22 Edwards Road, Burdett Road, Mile End. |
| (Western) | Mr. M. Whitfield, 32 Beaumont Square, Mile End. |
| (Northern) | Mr. R. C. Whayman, 18 Entick Street, Railway Place, Cambridge Road, Mile End. |
| Workhouse Master— | *Mr. E. Fox*, Bancroft Road, E. |
| **St. Olave's,** No. 1 ...... | Mr. J. H. Foreman, Steel Cottage, Steel Yard, London Bridge. |
| No. 2 ...... | Mr. W. H. Thomas, 1 Potters Fields, Horselydown. |
| No. 3 ...... | Mr. W. Fowle, 12 Longley Street, Bermondsey. |
| No. 4 ...... | Mr. G. C. Channon, 71 Layard Street, Bermondsey. |
| No. 5 ...... | Mr. W. Sanderson, 2, Gomm Road, Rotherhithe. |
| No. 6 ...... | Mr. F. C. Goodwin, 149 Abbeyfield Road, Rotherhithe. |
| Workhouse Masters— | *Mr. J. Norden*, Parish Street, Tooley Street, S.E. |
| | *Mr. W. Parkinson*, Tanner Street, Bermondsey, S.E. |
| **Paddington** (North) | Mr. J. E. Freeth, 69 Thorngate Road, W. |
| (South) | Mr. H. Vickerman, 2 Thorngate Road, W. |
| (West) | Mr. C. B. Nichols, 22 Woodfield Road, W. |
| Workhouse Master— | *Mr. E. Fowler*, Harrow Road, Paddington, W. |
| **St. Pancras—** | |
| No. 1 (North) | Mr. J. Stevens, 21 Chetwynd Road, N.W. |
| No. 1 (South) | Mr. H. T. Kobelt, 46 Maitland Park Road |
| No. 2 ......... | Mr. H. Payton, 167, Prince of Wales Road. |
| No. 3 ......... | Mr. W. Wheatley, 77 Patshull Road, N.W. |
| No. 4 ......... | Mr. Charles Tayler, 93 Albert St. |
| No. 5 ......... | Mr. J. E. Lake, 42 Robert Street. |
| No. 6 ......... | Mr. G. J. Moon, 28 Charrington St. |
| No. 7 ......... | Mr. H. H. Woodwell, 19A Fitzroy Square. |
| No. 8 ......... | Mr. J. Wright, 19 Harrison Street, Gray's Inn Road, W.C. |
| Workhouse Master— | *Mr. T. Miller*, King's Road, N.W. |

There were tents at Finchley, belonging to St. Pancras, but it is not certain that they are still there.

| Name of Union and No. of District. | Relieving Officer and Address. |
|---|---|
| **Poplar** (East)............ | Mr. B. Webster, 21 Aberfeldy Street, Bromley. |
| (West) ......... | Mr. F. Webster, 22 Woodstock Rd. |
| (Bromley)...... | Mr. J. C. Spratley, 153 Campbell Road. |
| (South) ......... | Mr. B. T. Wilson, 4 College View, North Greenwich, Poplar, E. |
| (Middle) ...... | Mr. M. Shorricks, 28 Hind Street, Stainsby Road. |
| (Bow)............ | Mr. S. Harvey, 23 Fairfield Road, Bow, E. |
| Workhouse Master— | *Mr. A. Deason*, High St., Poplar, E. |
| **St. Saviour's,** No. 1... | Mr. J. C. Glover, 61 Nelson Sq. |
| No. 2... | Mr. H. E. Williams, 117 Union Street. |
| No. 3... | R. T. Wooff, 15 Trinity Square. |
| No. 4... | Mr. R. Clunie, 38 Trinity Square. |
| No. 5... | Mr. W. J. Lyle, 9 St. George's Circus. |
| No. 6... | Mr. J. Smith, 51 Falmouth Road. |
| No. 7... | Mr. J. Whitham, 190 Westmoreland Road. |
| No. 8... | Mr. S. Bassett, 135 Grosvenor Pk. |
| No. 9... | Mr. J. W. Monk, 95 Smyth's Rd. |

| Name of Union and No. of District. | Relieving Officer and Address. |
|---|---|
| St. Saviour's—(*continued*) | |
| Workhouse Masters— | *Mr. E. Weekly*, Christchurch, Marlborough Street, Southwark. |
| | *Mr. W. Tompson*, Mint Street, Southwark, S.E. |
| | *Mr. R. Brown*, Newington Workhouse. |
| **Shoreditch**, No. 1...... | Mr. G. Mummery, 68 New North Road. |
| No. 2...... | Mr. W. G. Judge, 17 Hemsworth St. |
| No. 3...... | Mr. W. Glover, 45 Brownlow Road, Dalston. |
| No. 4...... | Mr. C. Kimber, 47 Huntingdon Street, Kingsland Road. |
| Workhouse Master— | *Mr. R. J. Larcombe*, Reeves Place, Hoxton, E. |
| **Stepney**, No. 1 ......... | Mr. J. Jones, 268 Burdett Road, Limehouse. |
| No. 2 ......... | Mr. T. G. Stacey, 95 Bromley Street, Ratcliffe, E. |
| Workhouse Master— | *Mr. C. Wellard*, St. Leonard Street, Bromley, E. |
| **Strand** ...................... | Mr. F. Wright, the Union, 6 Bow Street, Covent Garden. |
| Workhouse Master— | *Mr. J. H. Roach*, Edmonton, N. |
| **Wandsworth and Clapham**— | |
| Battersea West... | Mr. T. Barnes, Relief Station, Latchmere Road, Battersea. |
| Battersea East... | Mr. T. Tugwell, Relief Station, Latchmere Road, Battersea. |
| Wandsworth and Clapham—(*cont.*) | |
| Clapham ......... | Mr. W. J. Lyle, 21, Rozel Road, Clapham. |
| Putney ............ | Mr. E. Grafton, Stratford Gr. Putney |
| Streatham and Tooting Gaveney | Mr. A. Taylor, Merton Road, Lower Tooting. |
| Wandsworth...... | Mr. L. C. T. Darley, 1, Champion Ter., St. Ann's Hl., Wandsworth. |
| Workhouse Master— | *Mr. J. Hodge*, Garrett Lane, St. John's Hill, Wandsworth, S.W. |
| **Westminster**— | |
| Eastern ......... | Mr. J. H. Madge, 53, Dean Street, Soho, W. |
| Western......... | Mr. T. R. Warren, 52 Wardour St. |
| Workhouse Master— | *Mr. W. V. Minter*, Poland Street, W. |
| **Whitechapel**, North | Mr. J. C. McDonald, 2 Norton Folgate, E. |
| South | Mr. J. Eagles, 20, Mount Street, Whitechapel. |
| Workhouse Master— | (*Vacant*) S. Grove, Mile End Rd., E. |
| **Woolwich**— | |
| No. 1 (West Woolwich) | Mr. J. Moore, The Dispensary, Rectory Place, Woolwich. |
| No. 2 (East Woolwich) | Mr. A. J. Godfrey, Brewer Street, Woolwich. |
| No. 3 (Charlton) ...... | Mr. R. Padgham, Fair View Cottage, Old Charlton. |
| No. 4 (Plumstead)...... | Mr. H. Smith, Plumstead. |
| Workhouse Master— | *Mr. J. E. Rigden*, Plumstead. |

